# A role for platelets in metabolic reprogramming of tumor-associated macrophages

**DOI:** 10.3389/fphys.2023.1250982

**Published:** 2023-08-24

**Authors:** Ying Kang, Emmanuel Boadi Amoafo, Philomena Entsie, Gregory L. Beatty, Elisabetta Liverani

**Affiliations:** ^1^ Department of Pharmaceutical Sciences, School of Pharmacy, College of Health and Human Sciences, North Dakota State University, Fargo, ND, United States; ^2^ Department of Medicine, Division of Hematology-Oncology, Perelman School of Medicine, University of Pennsylvania, Philadelphia, PA, United States; ^3^ Abramson Cancer Center, Perelman School of Medicine, University of Pennsylvania, Philadelphia, PA, United States

**Keywords:** platelet, tumor microenvironment, tumor-associated macrophages, metabolic reprogramming, cancer

## Abstract

Cancer incidence and mortality are growing worldwide. With a lack of optimal treatments across many cancer types, there is an unmet need for the development of novel treatment strategies for cancer. One approach is to leverage the immune system for its ability to survey for cancer cells. However, cancer cells evolve to evade immune surveillance by establishing a tumor microenvironment (TME) that is marked by remarkable immune suppression. Macrophages are a predominant immune cell within the TME and have a major role in regulating tumor growth. In the TME, macrophages undergo metabolic reprogramming and differentiate into tumor-associated macrophages (TAM), which typically assume an immunosuppressive phenotype supportive of tumor growth. However, the plasticity of macrophage biology offers the possibility that macrophages may be promising therapeutic targets. Among the many determinants in the TME that may shape TAM biology, platelets can also contribute to cancer growth and to maintaining immune suppression. Platelets communicate with immune cells including macrophages through the secretion of immune mediators and cell-cell interaction. In other diseases, altering platelet secretion and cell-cell communication has been shown to reprogram macrophages and ameliorate inflammation. Thus, intervening on platelet-macrophage biology may be a novel therapeutic strategy for cancer. This review discusses our current understanding of the interaction between platelets and macrophages in the TME and details possible strategies for reprogramming macrophages into an anti-tumor phenotype for suppressing tumor growth.

## Introduction

Cancer incidence and mortality are rapidly growing around the world despite increased public awareness of cancer-related lifestyle factors and the application of early screening and diagnosis. To date, optimal treatments are lacking for many patients independent of cancer type (Wu and Dai, 2017). Recent advancements have underscored the close interaction between cancer cells and the immune system as well as the potential to harness the immune system for therapeutic benefit. However, cancer cells evolve vto evade immune surveillance and can promote immune suppression (Karachaliou et al., 2015). Together, these observations have culminated in immunotherapy as a standard of care for the treatment of cancer.

Macrophages are cells of the innate immune system that play a major role in inflammation and inflammatory-related diseases (Mills, 2012), including cancer (Liu et al., 2022). Macrophages differentiate from bone marrow derived monocytes upon activation and extravasation into tissues. However, populations of macrophages are also present in tissues as sentinel cells. These resident macrophages are derived embryonically rather than arising from the bone marrow (Liu et al., 2022). Depending on their surrounding microenvironment, macrophages can acquire a range of phenotypes. In the tumor microenvironment (TME), macrophages are classified as tumor-associated macrophages (TAM) (Mazzone et al., 2018). TAMs typically are engendered with an immunosuppressive phenotype. Given their fundamental role in pathophysiology, macrophages have been identified as promising targets for therapeutic strategies. Among the various strategies investigated to date, the most promising approaches have involved the depletion of macrophage subsets or reprogramming of the macrophage phenotype from pro-tumor to anti-tumor.

Both the tumor microenvironment and cell metabolism enable the TAM immunosuppressive phenotype. For example, modulation of the tumor microenvironment in which macrophages reside influences their phenotypical polarity and can shift their differentiation toward an anti-tumor phenotype. Identifying targets that can reprogram TAMs from pro-to anti-tumor (e.g., by shifting their cell metabolism or enhancing their phagocytic phenotype) is a promising approach being investigated to prevent and intervene on tumor growth.

Beyond macrophages, other determinants shape the tumor microenvironment and the growth of cancer. For instance, platelets are present in the TME and contribute to the tumor growth (Palacios-Acedo et al., 2019). Platelets have been shown to communicate with immune cells such as macrophages, neutrophils, and T lymphocytes (Stoiber and Assinger, 2020). Through the secretion of immune mediators (e.g., TGF-β diminishing natural killer (NK) cell anti-tumor activity) and cell-cell interaction (e.g., platelets coating around cancer cells), platelets contribute to immune suppression and support cancer cell evasion of immune surveillance (Braun et al., 2021). In other diseases, altering platelet secretion and cell-cell communication can reprogram macrophages and ameliorate inflammation (Carestia et al., 2019). Thus, this strategy might also be implemented in the TME.

In this review, we discuss our current understanding of the interaction between platelets and macrophages in the TME and detail possible strategies for reprogramming macrophages with an anti-tumor phenotype with the aim to suppress tumor growth.

## Tumor microenvironment

**FIGURE 1:**
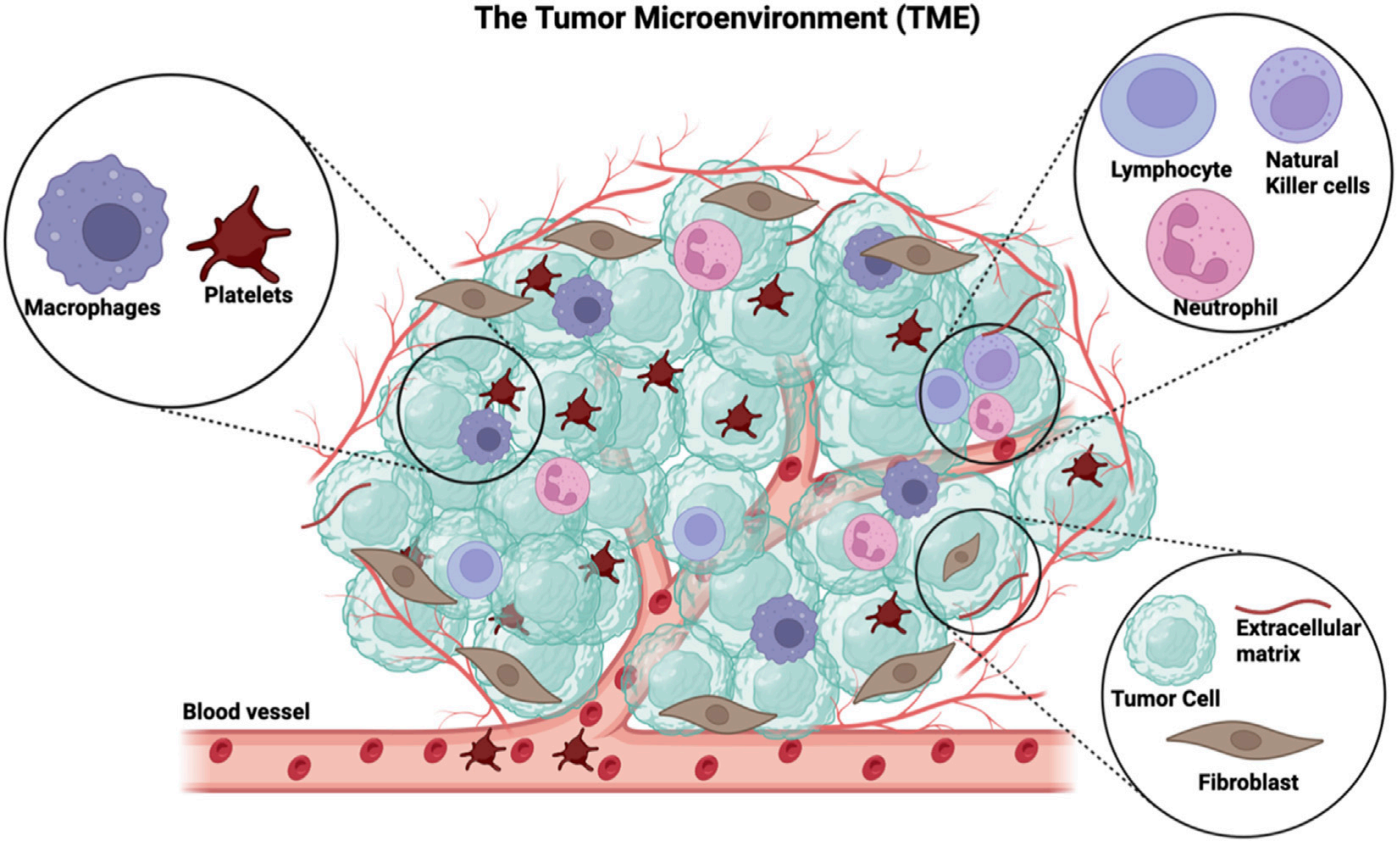
The general components of the tumor microenvironment. The TME comprises immune cells such as platelets, macrophages, lymphocytes, neutrophils, and natural killer cells. The non-immune part of the TME includes cancer cells, fibroblasts, extracellular matrix, and recently formed blood vessels. This figure has been generated using the Biorender software (https://www.biorender.com).

The tumor microenvironment (TME) is embodied by not only cancer cells but also the tissue that surrounds cancer cells within a tumor (Quail and Joyce, 2013) (Figure 1). This microenvironment is orchestrated by cancer cells and involves the induction of molecular, cellular, and physical changes within host tissues that ultimately support tumor growth (Baghban et al., 2020). The TME is a complex entity that consists of a heterogeneous population of cancer cells, resident host cells, secreted factors, and the extracellular matrix. (Hanahan and Coussens, 2012) (Figure 1). Characteristics of the TME vary between tumor types, but certain similarities exist, such as the presence of (i) immune and stromal cells (e.g., fibroblasts) (Candido and Hagemann, 2013), (ii) blood vessels, and (iii) extracellular matrix (Nallanthighal et al., 2019). Once established, the TME is maintained and co-evolves with the cancer cells (Wu and Dai, 2017; Baghban et al., 2020). Among the stromal cells present in the TME, immune cells are a predominant component and include T lymphocytes, macrophages, neutrophils, NK cells, and platelets (Heintzman et al., 2022) (Figure 1). The biology of infiltrating immune cells is influenced by cancer cells and contributes to the maintenance of the TME and fostering an environment that is supportive of tumor growth (Heintzman et al., 2022).

## Platelets in cancer

Platelets are anucleated cells indispensable for maintaining vascular integrity and hemostasis (Zamora et al., 2021), but also contribute to the inflammation (Liverani et al., 2014). For example, sufficient platelet counts, as well as adequate platelet functions, are required for the prevention of bleeding and to maintain a physiological inflammatory response (Chen et al., 2020). Consistent with this, recent studies have highlighted the importance of platelets in the cancer (Wang et al., 2022a).

Cancer patients can have an increased risk of thrombosis as high as 20% (Mosaad et al., 2021). Cancer cells actively secrete ADP, thromboxane, and thrombin (Jiang et al., 2017), which are molecules involved in platelet activation. Cancer-induced platelet activation is thought to be one reason why increased thrombosis is observed in cancer patients (Mosaad et al., 2021). Activated platelets contribute to cancer growth and metastasis (Morris et al., 2022). First, activated platelets can secrete angiogenic growth factors (Jiang et al., 2017), such as vascular endothelial growth factor (VEGF) and platelet-derived growth factor (PDGF). These molecules promote new blood vessel formation and thus, contribute to the delivery of nutrients to the cancer cells (Jiang et al., 2017). Second, platelets contribute to the formation of an immune suppressive milieu in the TME and its maintenance (Haemmerle et al., 2018).by secreting chemokines that can recruit macrophages into the TME. Third, platelets can secrete immune mediators such as CD40L, TGF-β, and programmed death-ligand 1 (PD-L1) (Liverani et al., 2014). These molecules can impair immune cell activation and in doing so, assist cancer cells in evading immune elimination (Candido and Hagemann, 2013).

Platelets have been shown to actively participate in the process of metastasis (Lucotti and Muschel, 2020). For instance, platelets can shield cancer cells from shear stress and immune recognition as they transit through the bloodstream and disseminate in distant organs. Specifically, cancer cells that detach from a primary tumor and intravasate into blood vessels become circulating tumor cells (CTC) (Bourcy et al., 2016). CTCs are usually low in numbers and if they can avoid immune elimination, they will ultimately become lodged in a distant tissue, adapt to a supportive niche and culminate in the metastasis (Bourcy et al., 2016). However, once in the bloodstream, there is a high probability that CTCs will be destroyed by high stress exerted by the blood flow and by the immune system. To this end, platelets may bind to CTCs and in doing so, form a platelet shield around CTCs that may protect CTCs from shear stress by reducing the exerted force (Lucotti and Muschel, 2020). Moreover, this platelet-CTC interaction can prevent the immune system from recognizing cancer cells and in doing so, promote immune evasion (Anvari et al., 2021). Further, platelets surrounding cancer cells can provide adhesive sites to the wall of blood vessels so that cancer cells may extravasate into tissues and give rise to metastasis. Thus, platelet interactions with cancer cells are instrumental in the metastatic cascade, offering protection, immune evasion, and facilitating extravasation into tissues.

A decrease in cancer growth has been observed upon platelet depletion and it has been specifically associated with a decrease in platelet secretion (Karachaliou et al., 2015). For instance, the anti-platelet drug ticagrelor synergized with gemcitabine significantly reduced tumor growth *in vivo* (Elaskalani et al., 2020; Palacios-Acedo et al., 2021), and the anti-platelet drug clopidogrel reduced tumor growth by itself *in vivo* (Elaskalani et al., 2020; Palacios-Acedo et al., 2021). Interestingly, platelet depletion can also inhibit metastasis formation. For instance, nuclear factor erythroid-derived 2 knockout mice that naturally lack platelets have shown a decrease in metastasis formation (Camerer et al., 2004). Similar results were observed when mice were platelet depleted using the anti-GP1bα antibodies (Labelle et al., 2014).

Taken together the data suggest that modulating platelet activation could be an effective strategy to diminish cancer growth and metastasis and to reprogram the immune system toward an anti-tumor phenotype.

## Tumor-associated macrophages (TAM)

**FIGURE 2:**
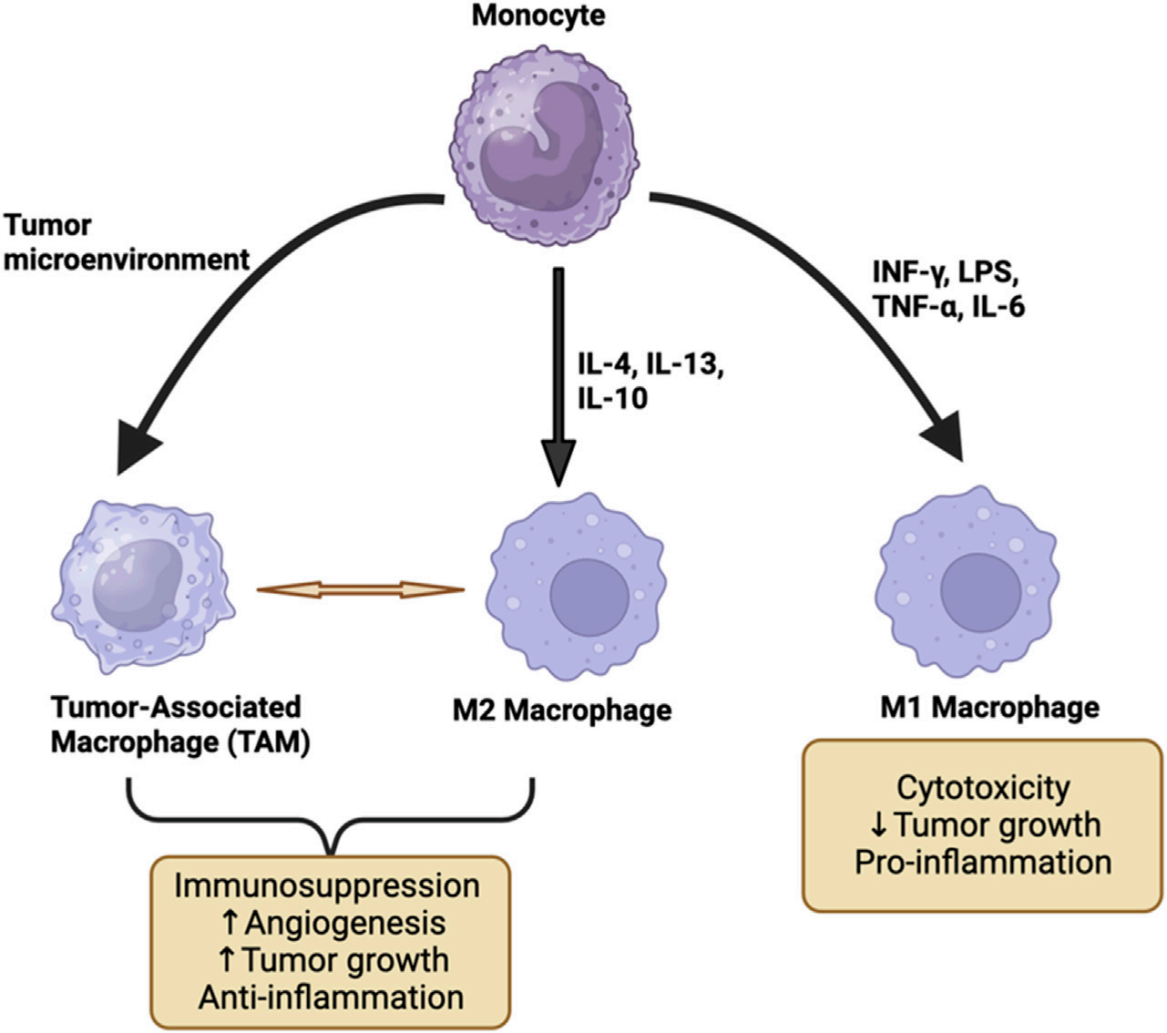
The differentiation of monocytes to M1, M2, and TAM macrophage phenotypes. M1 macrophages are activated by TNF, LPS, INF-γ, and IL-6 which exert pro-inflammatory effects and may result in the suppression of tumor growth. M2 and TAM are activated by IL-4, IL-13, and IL-10 and they promote tumor growth by suppressing the immune system and promoting angiogenesis. This figure has been generated using the Biorender software (https://www.biorender.com).

Macrophages exhibit polarization into a spectrum of different phenotypes in response to various activation stimuli present within their surrounding microenvironment (Mills, 2012). Two phenotypes based on *in vitro* polarizing stimuli have been defined to characterize macrophages as anti-tumor (M1) or pro-tumor (M2) (Figure 2). Macrophages that are activated by LPS and IFN-γ are called M1. M1 differentiation associates with iNOS expression resulting in production of nitric oxide (NO) and reactive oxygen intermediates (ROI). LPS exposure upregulates pro-inflammatory genes that are positively correlated with increased IL12/arginase ratio (Orecchioni et al., 2019). M1 macrophages also acquire a phenotype that has direct tumoricidal activity and promotes a T helper (T_H_) 1 immune response (Luheshi et al., 2014b) (Figure 2). In contrast, macrophages that are activated by IL-4 and IL-13 are identified as M2. IL-4 exposure increases the expression of the macrophage mannose receptor, which is normally downregulated by IFN-γ. M2-producing stimuli upregulate genes that correlate with a decreased IL12/arginase ratio (Buscher et al., 2017). The M2 macrophage phenotype suppresses a T_H_1 immune response and promotes angiogenesis (Luheshi et al., 2014b) (Figure 2). Based on these characteristics, the M1 macrophage phenotype supports inflammation and suppresses tumor growth, whereas the M2 macrophage phenotype suppresses an anti-tumor immune response and promotes tumor growth (Luheshi et al., 2014b). However, while this simplified characterization of macrophages is useful conceptually, within the TME, macrophages may be engendered with a range of phenotypes thereby illustrating the spectrum of macrophage biologies observed present in cancer (Heusinkveld et al., 2011) (Figure 2).

The presence of macrophages within tumors is fueled largely by bone marrow-derived monocytes but also is composed of resident tissue macrophages. Cancer cells recruit monocytes from the circulation into the TME by secreting specific cytokines such as CCL2, CCL11, CCL16, and CCL21 (Korbecki et al., 2020). Once monocytes reach the TME, the conditions of anoxia, inflammation, and increased lactic acid promote cell differentiation into TAMs (Penny et al., 2016). This macrophage subgroup has unique characteristics, although they can behave similarly to M2 macrophages (Quail and Joyce, 2013) (Figure 2). For instance, TAMs promote angiogenesis and tumor invasion and contribute to immune suppression (Quail and Joyce, 2013). Moreover, they secrete cytokines such as CCL2 to recruit other monocytic cells as well as cytokines such as IL-6, IL-8, and IL-10 that promote tumor growth (Wu and Dai, 2017).

## Metabolic reprogramming of macrophages in the tumor microenvironment

Differences in metabolism have been noted between macrophage phenotypes (Wang et al., 2022b). For instance, M1 macrophages demonstrate increased aerobic glycolysis as compared with decreased oxidative phosphorylation (OXPHOS) and fatty acid oxidation (FAO) (Viola et al., 2019). Glycolysis is important to support the high pro-inflammatory functions of M1 macrophages and is fundamental to maintaining the M1 phenotype (Koo and Garg, 2019). On the other hand, M2 macrophages utilize FAO and OXPHOS preferentially to maintain their phenotype (Liu et al., 2022), although M2 may also utilize glycolysis for energy. As cancer cells have high energy requirements and are the primary consumers of glucose in the TME (Jang et al., 2013), this competition for nutrients causes TAMs to shift toward OXPHOS and FAO metabolism (Mills, 2012; Liu et al., 2022). This metabolic propensity contributes to the maintenance of TAMs with an immunosuppressive phenotype (Dymicka-Piekarska et al., 2021). Further, upon hypoxic conditions, TAMs promote neoangiogenesis and tumor metastasis by shifting towards oxidative metabolism with decreased glycolysis through activation of mTOR signaling pathway (Magaway et al., 2019). In addition, within the TME, TAMs and cancer cells suppress T cells by metabolizing amino acids (e.g., arginine and tryptophan). For instance, myeloid cells in the TME can deplete stores of arginine through the expression of arginase (Kung et al., 1977). In human glioblastoma, TAMs demonstrate gene upregulation in the synthesis and transport of glutamine. There is also a reduction in tumor growth and infiltration of TAMs when amino acids are restricted.

The metabolic adaptation of macrophages relates intimately to their differentiation and therefore, their behavior (Zhang et al., 2021). Macrophages undergo metabolic reprogramming in the TME which affects their differentiation status (Wang et al., 2022b). In essence, metabolic reprogramming of TAMs contributes to retaining their immunosuppressive phenotype (Mazzone et al., 2018). The condition that causes this differentiation is a combined effect of hypoxia and cytokines (e.g., IL-6 and TGF-β) in the TME (Quail and Joyce, 2013; Wang et al., 2022b; Liu et al., 2022). For instance, hypoxia increases arginase-1 and mannose receptor (CD206) levels in TAMs (Kang et al., 2022). TAMs present in these hypoxic regions also induce expression of HIF-1α which induces a switch to glycolytic fermentation (Liu et al., 2022). Furthermore, cancer cell-derived lactic acid in the TME stabilizes the expression of HIF-1α under hypoxic conditions (Kang et al., 2022). These conditions cause a re-polarization of macrophages from an M1 phenotype to an M2 phenotype which is aided by lactic acid and characterized by increased arginase-1 level, CD206, and VEGF levels (Kang et al., 2022). As cancer cells maintain hypoxia and increased lactic acid content in the TME, this environment drives macrophages to assume a pro-tumor phenotype and enforces the polarization of newly recruited macrophages in the TME (Henze and Mazzone, 2016).

## Therapeutic strategy for metabolic reprogramming of macrophages

As TAMs promote tumor growth (Liu et al., 2022), two major strategies have been developed to intervene therapeutically in TAMs (Long et al., 2019). First, reducing the number of TAMs has been studied as an approach to convert tumors from immunotherapy-resistant to -sensitive (Omstead et al., 2022). Further, the depletion of TAMs may overcome chemotherapy resistance (Magkouta et al., 2021). For example, Colony-Stimulating Factor Receptor 1 (CSF1R) inhibitor was able to overcome PD-1/PD-L1 chemotherapy resistance in some cancers (Magkouta et al., 2021; Omstead et al., 2022). Second, strategies to reprogram TAMs with an anti-tumor phenotype are being explored, some of which target vascular biology and platelets, and are further discussed below (Liu et al., 2022).

Some cancer therapies target macrophages by depleting them. For instance, trabectedin selectively depletes monocytes and TAMs in patients and mice (Germano et al., 2013). However, other drugs have been tested for macrophage reprogramming. For example, therapeutics that affect vascular biology, such as anti-VEGFA and anti-Angiopoietin peptide 2 (ANGPT2), have been studied for their impact on macrophage biology (Kloepper et al., 2016). Specifically, these drugs not only aim to suppress angiogenesis but also inhibit glycolysis in the TME. The inhibition of glycolysis influences TAM biology and may contribute to metabolic reprogramming toward an anti-tumor phenotype (Kloepper et al., 2016). Moreover, the inhibition of glycolysis can inhibit cancer cell metabolism and therefore prevent cancer growth directly. In addition, therapeutics that restrict amino acid availability can regulate macrophage reprogramming. For example, arginase inhibitors that lower blood levels of arginase may increase the efficacy of anti-PD1 drugs (Badeaux et al., 2021). An increase in immunotherapy response with arginase inhibition is also observed in renal cell carcinoma and prostate tumor models (Grzywa et al., 2020). Genetic and pharmacological inhibition of glutamine, the most abundant amino acid in circulation, has also been shown to suppress tumor metastasis where pro-tumoral macrophages are converted to a pro-inflammatory state (Palmieri et al., 2017). Wu et al. discovered that glutamine restriction can provide an immunotherapeutic strategy for cancer. Polarization of macrophages to TAMs can also be promoted by succinate, which is actively secreted by cancer cells, identifying succinate availability as a potential target for the treatment (Wu et al., 2020).

Another approach involves the use of statins, drugs used worldwide to manage dyslipidemia but recently repurposed for cancer (Jiang et al., 2021). Statins are inhibitors of the hydroxymethylglutaryl-CoA reductase enzyme and statin treatment can lower total cholesterol, low-density lipoprotein, and triglyceride concentrations while increasing high-density lipoprotein (HDL) concentrations. The anticancer properties of simvastatin have been demonstrated to attenuate macrophage-mediated gemcitabine resistance (Xian et al., 2017; Jiang et al., 2021) and re-polarize TAMs (Jin et al., 2019). *In vitro*, studies have shown that treatment with simvastatin can promote macrophage polarization towards an M2 phenotype (Zi et al., 2021). Simvastatin can also decrease liver X receptors (LXR)/ATP-binding cassette transporter A1 (ABCA1) which is a receptor that regulates cholesterol homeostasis in macrophages (Zi et al., 2021). LXR is indeed required for M2 polarization. Moreover, simvastatin treatment also decreases *TGF-*β secretion (Zi et al., 2021).

In summary, these data suggest that targeting the metabolic reprogramming of macrophages to treat cancer may be a promising therapeutic strategy. So far, several drugs have been developed for macrophage reprogramming but further studies are required to identify more effective strategies that are targeted to specific cancers.

## Crosstalk between platelets and macrophages in the tumor microenvironment

The crosstalk between platelets and monocytes or macrophages has been established in inflammatory diseases such as sepsis (Carestia et al., 2019), kidney injury (Wen and Crowley, 2020), and fibrosis (Hoeft et al., 2023). This biology occurs due to the secretion of inflammatory mediators such as PF-4 or miRNA that promote macrophage differentiation (Scheuerer et al., 2000; Zhou et al., 2016). However, platelets may also alter macrophage polarization through the release of microparticles or exosomes (Heijnen et al., 1999). It has been shown that microparticles generated from platelets contain RANTES, MIF, CXCL-12, and IFN-γ that can promote the differentiation of monocytes into an M1 macrophage phenotype (Feng et al., 2019). In contrast, another study found that microparticles originating from apoptotic platelets polarized macrophages into an M2 macrophage phenotype (Vasina et al., 2011), suggesting that platelet age may influence how microparticles from platelets impact macrophage biology. Platelet-derived exosomes may also inhibit the activation of NLPR3 inflammasome and in doing so, promote an M2 phenotype (Qian et al., 2022). Exosomes from platelets also contain inflammatory mediators and chemokines such as PF-4, RANTES, and CXCL3 (Wei et al., 2022). Together, these data show that platelets can promote macrophage differentiation through shedding of microparticles or exosomes but further studies are needed to fully understand the impact of platelet biology on macrophage phenotype. Further, whether platelet-macrophage crosstalk occurs strictly through secretion of molecules or cell-cell interactions is also still unclear.

Crosstalk between platelets and macrophages has also been shown to be important for the progression of cancer. One study showed that anti-platelet drug clopidogrel promoted an anti-tumoral hepatic macrophage phenotype (Pavlović et al., 2021). Despite this, no information is available on how platelet secretion or cell interaction contributes to maintaining the polarization of macrophages in the TME. However, as mentioned above, both platelets and TAM are present in the TME and they both appear to contribute to tumor growth and immune suppression raising a compelling hypothesis that their interaction may be a determinant of tumor biology (Baghban et al., 2020). Below, we address several molecular interactions that may occur between platelets and macrophages.

## PD-1/PD-L1

Programmed cell death protein 1 (PD-1) is a cell surface receptor present on immune cells such as activated T cells, NK cells, B Lymphocytes, dendritic cells, and macrophages (Agata et al., 1996). Under physiological conditions, PD-1 can reduce cell activation and maintain immune tolerance (Agata et al., 1996). However, activation of PD-1 is also an immune suppressive strategy exploited by cancer to evade immune elimination (Acurcio et al., 2022). PD-1 ligand (PD-L1) is a transmembrane protein expressed by macrophages, activated T cells, B cells, DCs, epithelial cells, and platelets (Zhang et al., 2018). PD-L1 can also be expressed by cancer cells and represents an adaptive immune escape mechanism. Once bound to its receptor (PD-1), PD-L1 negatively regulates immune cell functions. As a result, cancer cells that acquire PD-L1 expression demonstrate increased resistance to cancer immunotherapy (Rolfes et al., 2018). PD-1/PD-L1 checkpoint inhibition suppresses PD-L1 negative tumors by interfering with platelet PD-L1 binding, and inhibition in PD-L1 negative tumor growth has been observed upon platelet depletion associated with a decrease in platelet PD-L1 secretion (Zaslavsky et al., 2020). Several other studies have also shown that drugs that inhibit the PD-1/PD-L1 axis can promote immune surveillance and as a result, reduce tumor growth. Indeed, anti-PD1/PD-L1 immunotherapy has demonstrated remarkable clinical efficacy in a variety of cancers.

## CD40/CD40L

CD40 is a tumor necrosis factor receptor superfamily member (Grewal and Flavell, 1998) and is widely expressed by immune cells including B cells and macrophages (Grewal and Flavell, 1998). CD40 engages with the CD40 ligand (CD40L) and via this interaction leads to the activation of macrophages and the “licensing” of dendritic cells with potent T cell priming properties (Beatty et al., 2017). CD40 activation has been shown to stimulate productive T cell immune surveillance in cancer leading to inhibition of tumor growth as seen in animal models as well as in some patients (Quezada et al., 2004). CD40 is expressed on TAMs and activating this receptor through CD40 agonists can activate macrophages (Luheshi et al., 2014a). However, how TAMs respond to CD40 activation depends on the cytokine content of the TME. Different cytokines secreted from T_H_1 and T_H_2 cells can influence the effects of CD40L/CD40 activation (Peng et al., 1998; Snyder et al., 2007). For instance, a T_H_1-dominated tumor microenvironment can promote the ability of CD40-activated macrophages to kill malignant cells (Grewal and Flavell, 1998; Peng et al., 1998). On the other hand, in a T_H_2-dominated tumor microenvironment, CD40-activated macrophages fail to acquire an anti-tumor phenotype (Grewal and Flavell, 1998; Peng et al., 1998). Interestingly, the TME also suppresses cytotoxic T cell differentiation, while promoting regulatory T cells, suggesting that CD40L secretion might be a strategy to overcome the immunosuppressive elements of the TAM phenotype (Stirm et al., 2023). These data suggest that modulating CD40 signaling can repolarize macrophages from tumor-promoting to tumor-suppressive. Platelets significantly contribute to the concentration of soluble CD40L during inflammation (Cognasse et al., 2022) and in the TME. Activation of the CD40/CD40L pathways appears to promote the TAM phenotype. However, it is noteworthy that there is currently no evidence that soluble CD40L specifically from platelets alters macrophage polarization in the tumor microenvironment, although this is an important area for future investigation. Nonetheless, CD40 activation via agonists are an important strategy for altering macrophages to acquire an M1 phenotype that is anti-tumor (Long et al., 2016; Lim et al., 2022).

## TGF-β

Transforming growth factor-beta (TGF-β) is a ubiquitous, modulator of cellular responses under physiological conditions (Kubiczkova et al., 2012). However, the role of TGF-β is still unclear although it seems to contribute to maintaining an immunosuppressive environment. Production of TGF-β has been observed during diseases including cancer and high concentrations have been observed in the TME (Kim et al., 2021). Interestingly, platelets are a significant contributor to TGF-β content in the TME (Huong et al., 2019; Dymicka-Piekarska et al., 2021). Indeed, TGF-β contributes to regulatory T cell proliferation and therefore maintains an immunosuppressive environment (Colak and Ten Dijke, 2017). In cancer, activation of the TGF-β pathway enhances cell proliferation, migratory invasion, and metastatic spread within the tumor microenvironment and suppresses the immunosurveillance (Pickup et al., 2013). TGF-β can be secreted by macrophages and can act in a paracrine or autocrine manner by influencing the biology of other stromal cells (e.g., fibroblasts) in the TME or by signaling in macrophages as they also express the TGF-β receptor (Sun et al., 2021). In doing so, TGF-β can regulate numerous responses in monocytes and macrophages, such as activation, cytokine production, host defense, and chemotaxis (Kubiczkova et al., 2012). However, the effect of TGF-β receptor activation depends on the phenotype of macrophages (Kubiczkova et al., 2012). TGF-β induces polarization into the M2-like phenotype of macrophages by repressing the expression of pro-inflammatory TNF-α and IL-12 and increasing the expression of IL-10 (Zhang et al., 2016).

## PF-4

Platelet factor 4 (PF-4) is a cytokine that is released from alpha granules of activated platelets (Liverani et al., 2014). Overexpression of PF-4 has been observed in cancers such as lung cancer and correlates with decreased patient survival (Pucci et al., 2016). Overall, PF-4 has been identified as a cancer-enhancing molecule (Pucci et al., 2016) that alters bone marrow hematopoiesis and increases platelet accumulation in the TME. Interestingly, PF-4 can promote monocyte differentiation into macrophages *in vitro* (Scheuerer et al., 2000). PF-4-induced macrophages also appear to acquire a specific phenotype, that has characteristics of both M1 and M2 macrophages (Scheuerer et al., 2000). As a result, PF-4-activated macrophages have been classified as M4 macrophages: they secrete pro-inflammatory cytokines such as TNF, IL-6, MMP-7, and MMP-12 (de Sousa et al., 2018). No studies to date have investigated whether PF-4 might contribute to macrophage differentiation in the TME and/or to maintaining the TME phenotype.

## Podoplanin/CLEC-2

Podoplanin is a heavily glycosylated small transmembrane glycoprotein. It is consistently expressed in type I lung epithelial cells, fibroblastic reticular cells, lymphatic endothelial cells, and podocytes (Krishnan et al., 2018). It is upregulated on inflammatory macrophages (Including TAM), T helper 17 cells, fibroblasts, and cancer cells (Krishnan et al., 2018; Bieniasz-Krzywiec et al., 2019; Bourne et al., 2021). Podoplanin is the endogenous ligand of CLEC2, which is a hemi-immunoreceptor tyrosine-based activation motif (Hemi-ITAM) receptor. CLEC-2 is constitutively expressed on platelets (Navarro-Nunez et al., 2013) and a subset of dendritic cells (de Winde et al., 2018). Once activated, CLEC-2 activation induces platelet activation (Dangelmaier et al., 2022). CLEC-2 exists as a transmembrane protein as well as in its soluble form (Meng et al., 2021). Podoplanin expressed in cancer cells activates platelets through CLEC-2 (Suzuki-Inoue, 2018). Therefore, CLEC-2-podoplanin interactions can stimulate cancer-associated thrombosis (Hwang et al., 2021). Further, in the bloodstream, CLEC-2-podoplanin can promote cancer cell evasion of the immune surveillance (Hwang et al., 2021).

## ADP/receptor P2Y_12_

P2Y_12_ is a G-protein coupled receptor that is activated by ADP (Kim and Kunapuli, 2011). P2Y_12_ is essential for platelet function (Kim and Kunapuli, 2011), particularly secretion. However, P2Y_12_ has also been identified in immune cells (Entsie et al., 2023) and cancer cells (Hu and Zhang, 2023). P2Y_12_ is expressed in macrophages (Isfort et al., 2011; Pavlovic et al., 2020; Chen et al., 2022) but the effect of activating P2Y_12_ in macrophages is still largely unknown. A recent study revealed that P2Y_12_ plays a role in mediating macrophage polarization toward an M2 phenotype (Pavlovic et al., 2020). Antagonizing the receptor P2Y_12_ using ticagrelor, can also increase phagocytic activity in macrophages (Pavlovic et al., 2020). These data suggest that the P2Y_12_ blockade may then promote an anti-tumor phenotype (Pavlovic et al., 2020). Consistent with this, cancer cells actively secrete ADP in the TME. ADP can then activate platelets through P2Y_12_ and as a result, activated platelets will secrete ADP, further increasing ADP in the TME. Taken together, these data suggest that modulating ADP concentration in the TME might influence macrophage biology. Hence, blocking P2Y_12_ may be an approach for reprogramming macrophages, by interfering directly with macrophage activation, but also by decreasing the overall concentration of ADP from platelets.

## Conclusion and future direction

Targeting the metabolism-related pathways of TAMs represents a possible strategy for reprogramming macrophages with anti-tumor phenotype by promoting phagocytic and immune-stimulatory functions (Liu et al., 2019). To this end, platelets appear to influence macrophage differentiation and metabolic programming. In particular, among the immunomodulators secreted by platelets so far, CD40L and PD-L1 appear to be the most relevant for macrophage differentiation. Other molecules such as TGF- β, ADP, and PF-4 are also secreted by platelets in a significant amount in the TME. However, these molecules appear to crosstalk preferentially with T cells and neutrophils but may still contribute to macrophage differentiation in other inflammatory conditions. Further investigation is necessary to understand the impact of altering platelet function in the TME on macrophage biology. It is possible that intervening on platelet biology could be a novel strategy to induce TAMs to re-differentiate into an anti-tumor phenotype. To this end, modulating platelet biology may be a promising and novel strategy to prevent tumor growth. Anti-platelet therapy, such as ticagrelor, which is already under investigation is one approach. However, as discussed in this review, multiple new promising targets warrant further investigation.
